# Evaluating the Timing of Cholecystectomy in Gallstone-Induced Pancreatitis: Adherence to Guidelines and Impact on Patient Outcomes

**DOI:** 10.7759/cureus.95486

**Published:** 2025-10-27

**Authors:** Mohab Elsalahi, Rothana Mohammed, Waleed Alsanie, Fayrouz El Shawadfy, Mohamed Sabry, Beshr Mosa Basha, Mazin Karem, Shahla Shamshad, Moatasem Alaa Tawfik Askar, Youssef Mohamed Abdalla Hassan, Haged Mohamed, Alaa Elsayed, Mostafa Afifi, Ahmad Adel Abdelhameed Muhammad, Freya Dodgins, Amr M Soliman, Mennat Allah Oda, Abdulmabod Omar

**Affiliations:** 1 General Surgery, NHS Lanarkshire, Glasgow, GBR; 2 Pediatrics, Peterborough City Hospital, Peterborough, GBR; 3 Surgery, Dr. Hassan Ghazzawi Hospital, Jeddah, SAU; 4 Surgery, Badr Hospital, Cairo, EGY; 5 Surgery, Faculty of Medicine, Mansoura University, Mansoura, EGY; 6 General Surgery, Dr. Sulaiman Al Habib Hospital, Khobar, SAU; 7 Psychiatry, North Devon District Hospital, Devon, GBR; 8 Emergency Medicine and Critical Care, Dr. Hassan Ghazzawi Hospital, Jeddah, SAU; 9 Orthopedics, El Bagour Specialized Hospital, Menoufia, EGY; 10 Obstetrics and Gynecology, Jaber Al Ahmad Al Sabah Hospital, Kuwait, KWT; 11 Intensive Care Medicine, Al Tadamon Hospital, Port Said, EGY; 12 Internal Medicine, Al Tadamon Hospital, Port Said, EGY; 13 Clinical Pathology, Faculty of Medicine, Al-Azhar University, Cairo, EGY; 14 General Surgery, North Devon District Hospital, Devon, GBR; 15 Surgery, Demerdash Ain Shams University Hospital, Cairo, EGY; 16 Pediatric Surgery, Zagazig University Hospitals, Zagazig, EGY

**Keywords:** cholecystectomy, clinical audit, gallstone pancreatitis, guideline adherence, retrospective study, surgical timing

## Abstract

Background

To avoid recurrence, patients with mild to moderate gallstone-induced pancreatitis should have a cholecystectomy as soon as possible. The aim of this study was to ascertain if adherence to surgical time recommendations influences patient outcomes, especially regarding readmission rates.

Methodology

We performed a retrospective study of patients admitted between September 2024 and July 2025 with gallstone pancreatitis at Dr. Hassan Ghazzawi Hospital, Jeddah, Saudi Arabia. Comparing those treated before and after the implementation of guideline recommendations for early cholecystectomy. Demographic information, the number of admissions, and the timing of the operation were obtained from hospital records. Patients were divided into four categories: timely (index admission), moderately delayed (weeks), significantly delayed (months/years), and non-surgical. Continuous variables were summarized as means and standard deviations, whereas categorical variables were displayed as frequencies and percentages. The groups have been compared using Fisher's exact test as well as the Chi-square test. A p-value of <0.05 has been considered statistically significant.

Result

The study covered 47 surgical patients and 47 non-surgical patients. Following guideline adoption, the proportion of timely cholecystectomies increased (32% vs. 53%), while significantly delayed procedures dropped (39% vs. 7%), although this trend did not achieve statistical significance (p = 0.075). The rate of multiple admissions was not statistically different (19% vs. 13%, p = 1.0). In the non-surgical sample, the mean number of admissions reduced (1.57 to 1.41) while recurring admissions (>1) fell (40% to 29%). The mean patient age was similar between groups (57 and 58 years).

Conclusion

Guideline adherence improved surgical timing and decreased recurrence of gallstone pancreatitis. Early cholecystectomy, preferably during the initial admission, should be the standard of care. Non-surgical patients remain at increased risk of readmission and require regular monitoring.

## Introduction

Gallstone disease is the most common cause of acute pancreatitis, accounting for over half of all cases worldwide [1]. After a first incident of gallstone-induced (biliary) pancreatitis, patients remain at substantial risk of recurring biliary events (44.1%), including repeat pancreatitis, cholangitis, or biliary colic, unless effective therapy is delivered [2]. Open cholecystectomy is commonly regarded as the definitive operation to avoid recurrence since it removes the gallbladder and its stones, the source of biliary inflammation [3]. However, the best timing for cholecystectomy in individuals with gallstone pancreatitis has long been controversial [4]. While traditional management recommended postponing surgery until inflammation has subsided, emerging data supported an early surgical approach [5]. Several observational studies have found that early cholecystectomy (at index admission or within a short interval) is associated with lower recurrence rates, shorter hospital stays, and no significant increase in surgical complications [6].

As previously reported [6], a randomized prospective study involving patients with mild-to-moderate biliary pancreatitis indicated that performing cholecystectomy early did not lead to a significant rise in surgical complications or conversion rates among the patients. In contrast, the delayed group showed a markedly higher incidence of recurrent biliary events (44.1% vs. 0%; p ≤ 0.0001) and extended hospital stays (9 vs. 8 days; p = 0.002) [6]. Similarly, early cholecystectomy is safe and effective in selected patients with mild biliary pancreatitis; it reduces readmissions and shortens hospital stay [7]. Another randomized research comparing extremely early surgery (within 48 hours) to delayed intervention found that early cholecystectomy resulted in a considerably shorter hospital stay (mean 3.5 vs. 5.8 days; p = 0.0016) without affecting perioperative complications or conversion rates [8]. On the other hand, in a large registry cohort of over 17,000 patients, guideline-adherent cholecystectomy (during index hospitalization or shortly after) was associated with significantly lower rates of subsequent acute pancreatitis (3% vs. 13%) and chronic pancreatitis (1% vs. 4%) compared to non-adherent cases (P < 0.001) [9]. A meta-analysis of 11 randomized studies with 1,176 patients indicated that early cholecystectomy significantly lowers recurrent biliary events (RR 0.10, 95% CI 0.05-0.19) and recurrent pancreatitis (RR 0.21, 95% CI 0.09-0.51) without a statistically significant rise in operating complications [10].

Despite these findings and cholecystectomy guidelines, real-world adherence remains poor in many centers. Many hospitals continue to conduct cholecystectomies late or not at all, leading to unnecessary readmissions and consequences [9]. The Academy of Medical Royal Colleges guideline expressly suggests that patients with moderate gallstone pancreatitis or acute cholecystitis have an index admission laparoscopic cholecystectomy to decrease recurrence risk [3].

Dr. Hassan Ghazzawi Hospital now follows a guideline-adherent schedule for cholecystectomy procedures. In this context, it is vital to monitor operation scheduling, patient adherence to guidelines, and results in actual hospital settings. Such investigations serve to close the "evidence-to-practice" gap, highlight systemic impediments, and enable local performance benchmarking. Furthermore, they give data to help determine whether guideline implementation is genuinely associated with fewer readmissions and problems in a real-world patient population.

The purpose of this retrospective study is to assess surgical timing, recurrence (admissions with pancreatitis), and unfavorable outcomes before and after implementing the Academy of Medical Royal Colleges guideline. We aim to assess the impact of guideline-based practices on local communities and identify gaps in adherence to inform quality improvement efforts.

## Materials and methods

Study design

This was a retrospective study conducted at Dr. Hassan Ghazzawi Hospital, aiming to evaluate the timing of cholecystectomy in patients admitted with gallstone-induced pancreatitis. The study compared patient outcomes before and after adherence to national cholecystectomy guidelines [3], specifically the recommendations by the Academy of Medical Royal Colleges, which advise performing an index cholecystectomy during the initial admission for patients with mild gallstone pancreatitis.

Study population

The study includes 94 patients admitted between September 1, 2024, and July 31, 2025, who presented with gallstone-induced pancreatitis.

Guideline reference

Adherence was assessed against the guidelines published [3], which recommend index laparoscopic cholecystectomy during the same admission for patients with acute cholecystitis or mild gallstone pancreatitis to reduce complications.

Study period and setting

A total of 94 patients admitted with gallstone pancreatitis had data collected. Information collected included patient demographics (age, sex), whether cholecystectomy was performed, and its timing (categorized as timely/index admission, moderately delayed, or delayed), number of pancreatitis-related admissions (to assess recurrence), presence of interventions such as endoscopic retrograde cholangiopancreatography (ERCP), and mortality status during the study period. Patients were divided into two groups based on the timing relative to the hospital's adoption of guideline-compliant surgical practice: Group I, pre-guideline adherence group, and Group II, post-guideline adherence group.

Criteria for the study

The inclusion criteria for the study populations was as follows: (a) patients admitted to Dr. Hassan Ghazzawi Hospital between 01/09/2024 and 31/07/2025; (b) diagnosed with gallstone-induced acute pancreatitis, confirmed by clinical presentation, imaging, and/or laboratory results; (c) patients who underwent cholecystectomy during or after admission or managed conservatively (for comparison); and (d) patients with at least one documented hospital admission related to gallstone-induced pancreatitis during the study period.

The exclusion criteria for the study populations was as follows: (a) patients with non-gallstone-related pancreatitis (e.g., alcohol-induced, idiopathic); (b) patients who did not survive index admission due to unrelated causes; (c) patients unfit for surgery due to comorbidities; (d) incomplete records or missing follow-up data; (e) patients who underwent cholecystectomy before the index admission; and (f) patients transferred from or to other hospitals during the acute episode where timing of surgery could not be accurately tracked.

Exposure and outcome measures

Exposure measure composed of the timing of cholecystectomy, classified based on adherence to established guidelines (index admission surgery considered timely). Outcomes measure composed of rates of recurrent pancreatitis admissions, readmissions, and biliary complications in relation to surgical timing and mortality during follow-up.

Statistical analysis

Data were analyzed using IBM SPSS Statistics for Windows, version 25 (IBM Corp., Armonk, NY, USA). Continuous variables (e.g., age, number of admissions) were summarized as medians with ranges where appropriate or as means ± standard deviations (SD). Categorical variables (e.g., timing of surgery, number of admissions) were expressed as frequency and/or percentage. Comparisons between groups (before vs. after guideline implementation) were performed as follows: (a) admissions (single vs. ≥2): analyzed using Fisher’s exact test, and (b) timing of surgery (timely, moderately delayed, markedly delayed): analyzed using a Chi-square test for independence. A p-value < 0.05 was considered statistically significant during statistical analyses.

Ethical approval

The Institutional Ethics Committee, Dr. Hassan Ghazzawi Hospital, Jeddah, granted ethical permission (DHGH-MBEC-2507). Each method that involved human subjects was carried out in compliance with the Declaration of Helsinki [11]. Prior to data collection, all subjects provided written informed consent. Throughout the study, individuals' anonymity and confidentiality were rigorously protected.

## Results

A total of 47 selected individuals did not undergo cholecystectomy surgery. The average age of patients who did not undergo surgery was similar between groups (57.3 years before vs. 58.2 years after guideline adherence). The median age increased after the cholecystectomy, 2020 guideline [3] adherence (57.0 to 64.0), suggesting a slightly older population was managed conservatively post-guideline. The mean number of admissions decreased slightly from 1.57 to 1.41 after adherence to guidelines. There was a notable reduction in recurrent admissions among non-surgical patients from 40% before guideline adherence to 29.4% after (Table 1).

**Table 1:** Characteristics of patients managed without cholecystectomy surgery (pre- vs. post-guideline) SD: standard deviation

Parameter	Before guideline (n=30)	After guideline (n=17)
Mean age (years)	57.3	58.2
Median age (years)	57.0	64.0
Age range (years)	25–80	21–83
Mean admissions	1.57	1.41
Admissions SD	0.77	0.71
Recurrent admissions (%)	40.00%	29.40%

Outcomes in patients who underwent cholecystectomy surgery

A total of 47 patients underwent surgery: 32 patients before guideline adherence and 15 patients after. Two patients were eliminated from the timing study because their cholecystectomies were performed at other facilities, where timing and admission information were unavailable.

Before guideline adherence, the mean age was 53.7 years (median 55.5, range 23-92 years). The group was composed of 40.6% male and 59.4% female patients. After guideline adherence, the mean age was similar at 53.4 years (median 57.0, range 21-85 years), with a lower proportion of male patients at 26.7% and female patients comprising 73.3% of the cohort. There were no significant differences in mean age between the two groups. However, the post-guideline group had a higher proportion of female patients. Age does not appear to be a strong determinant of recurrence, given similar age distributions between recurrent and non-recurrent patients.

Patients were categorized based on the timing of their cholecystectomy into four groups: (a) timely (surgery during index admission), (b) moderately delayed (surgery within weeks), (c) delayed (surgery after several months), and (d) other (e.g., surgery in private practice or unclear timing).

Table 2 summarizes the number of admissions prior to cholecystectomy based on the timing of the surgery, both before and after the implementation of guideline adherence. Patients who received timely cholecystectomy (index admission or ERCP during the index admission) consistently required only one admission, both before and after the guidelines (1.00 ± 0.00 vs. 1.00 ± 0.00, p = 1.000). In the moderately delayed group, the average number of admissions increased following the guidelines (1.40 ± 0.55 vs. 1.00 ± 0.00), indicating a trend that did not achieve statistical significance (p = 0.090). Conversely, cases that experienced significant delays had a higher number of admissions prior to the guidelines (2.00 ± 0.74 vs. 1.00 ± 0.00), although this difference was not statistically significant (p = 0.539). In summary, the data indicate a greater variability and increased admission burden among delayed cases before adherence to the guidelines, while timely cases were consistently managed with a single admission. Following guideline implementation, the proportion of timely cholecystectomies increased (from 32% to 53%). At the same period, the proportion of moderately delayed procedures increased somewhat (29% to 40%), whereas notably delayed surgeries declined significantly (39% to 7%). This shift demonstrates a strong trend towards quicker surgical intervention after guideline implementation, with fewer patients having lengthy waits. These findings support the previously recognized role of early cholecystectomy in lowering the incidence of recurrent biliary episodes.

**Table 2:** Outcomes of patients managed with cholecystectomy (pre- vs. post-guideline) SD: standard deviation

Admissions (mean ± SD)	Before guidelines (n=32)	After guidelines (n=15)	U-value (Mann–Whitney U)
Timely (n=10/n=8)	1.00 ± 0.00	1.00 ± 0.00	1.000
Moderately delayed (weeks) (n=9/n=6)	1.00 ± 0.00	1.40 ± 0.55	0.090
Markedly delayed (months/years) (n=12/n=1)	2.00 ± 0.74	1.00 ± 0.00	0.539

Table 3 compares admissions and timing across groups. There was no significant difference in the proportion of patients who needed only one admission versus many admissions before and after the guidelines (19% vs. 13%, p = 1.000, Fisher’s exact). An analysis of surgical timing revealed a trend towards earlier interventions following adherence to guidelines, with a higher percentage of patients receiving timely surgeries (32% compared to 53%, p = 0.202). The proportion of moderately delayed cases was comparable (29% versus 40%, p = 0.508), while fewer patients faced significant delays (39% against 7%, p = 0.037). Nonetheless, this finding did not reach statistical significance (p = 0.075, Chi-square). Although the statistical tests yielded no significant results, the data indicate that guideline adoption was related to a considerable movement towards earlier cholecystectomy. Specifically, more patients had surgery at or shortly after their index admission, but the proportion of cases that were significantly delayed declined. The lack of significance (χ² p = 0.075) is most likely due to the limited sample size, although the observed trend confirms the clinical benefit of following guidelines. Importantly, there was no difference in the rate of repeated admissions between groups, indicating that guideline adherence had a greater impact on surgery time than overall admission burden.

**Table 3:** Comparison of admissions and timing of surgery before vs. after guideline adherence ¹ Fisher’s exact test; ² Chi-square test across three categories (timely, moderately delayed, markedly delayed)

Variable	Before guidelines (n=32)	After guidelines (n=15)	U-value
Admissions			
Single admission	26 (81%)	13 (87%)	1.000¹
≥2 admissions	6 (19%)	2 (13%)	1.000¹
Timing of surgery			
Timely	10 (32%)	8 (53%)	0.202¹
Moderately delayed (weeks)	9 (29%)	6 (40%)	0.508¹
Markedly delayed (months/years)	12 (39%)	1 (7%)	0.037¹
Chi-square test	U = 0.075²		

## Discussion

In our retrospective study at Dr. Hassan Ghazzawi Hospital, we discovered that the percentage of timely cholecystectomies increased following the implementation of the recommendations (from 32% to 53%), whereas significantly delayed procedures decreased (39% before vs. 7% after). Following guideline implementation, the mean number of pancreatitis admissions remained stable in the timely group but increased slightly in the moderately delayed group. In the non-surgical sample, mean admissions declined from 1.57 to 1.41, and the number of patients with recurring admissions (>1) dropped from 40% to 29%.

Given the demographic analysis (mean ages ~57 before vs. ~58 after), it is unlikely that a younger/healthier post-guideline cohort alone could account for the observed changes because patient age did not significantly differ between groups. These findings suggest that guideline-based scheduling may reduce long-term surgical delays and the burden of recurring admissions, even among nonoperatively treated patients. The demographic analysis (mean ages ~57 before vs. ~58 after) suggests that the observed changes cannot be attributed solely to a younger/healthier post-guideline cohort, as patient age did not differ substantially between groups.

However, the fact that repeated admissions occurred in the non-surgical group even after the guidelines were implemented emphasizes that patients are still at risk when they choose not to have surgery. In other words, adhering to guidelines seems to reduce, though not completely eliminate, recurrence in patients who have not undergone surgery, potentially due to some patients declining treatment or not qualifying for it. Two fatalities were reported in the pre-guideline cohort among patients who did not receive surgical intervention, suggesting a correlation between non-operative management and unfavorable results. Several individuals had ERCP during their initial admission without undergoing cholecystectomy. While ERCP treats bile duct obstruction, it does not eliminate the cause of gallstones and thus does not prevent recurrence. The guidelines advocate ERCP only for cases of cholangitis or ductal stones, not as a substitute for cholecystectomy. These findings indicate potential gaps in surgical planning or medical fitness problems, emphasizing the importance of integrated multidisciplinary care and follow-up.

We conclude that early (or index admission) cholecystectomy lowers recurrent biliary occurrences without raising complications. Along with a multicenter study conducted in the UK between 2006 and 2008, cholecystectomy performed within the recommended timeframes (index admission or within two weeks) was linked to a decreased recurrence risk [12]. For instance, early laparoscopic cholecystectomy was linked to fewer gallstone-related events and a shorter length of stay than delayed cholecystectomy without significantly increasing intraoperative or postoperative complications (RR for readmission ~0.17) in a meta-analysis of 19 studies (2639 patients) [13]. Similarly, early cholecystectomy in moderate gallstone pancreatitis was linked to shorter surgical time, shorter length of stay, and more concurrency of biliary procedures, but it was not linked to increased morbidity or death, according to research employing a multicenter database (National Surgical Quality Improvement Program (NSQIP)) [14]. A considerably lower hospital length of stay (median 58 hours vs. 167 hours) was also observed in the early surgery arm of Liu et al.'s research randomized study, with no increase in complications or readmissions [14]. Our data support these conclusions: mean admissions in the "timely" groups stayed at 1.00 with little variation, indicating limited recurrence and acceptable safety in our sample.

Consistent with countrywide research of gallstone pancreatitis in the US from 2016 to 2019, delayed cholecystectomy (>2 days) was independently linked to higher expenses, 30-day readmissions, and significant adverse events than early surgery (≤2 days) [15]. This supports the notion that delays carry real risks. However, there was a slightly greater, though not statistically significant, prevalence of surgical complications (13% vs. 6%). On the other hand, McDermott et al. concluded that guideline-concordant surgery reduced recurrence but must balance operative risk [16]. Another 10-year regional investigation on moderate gallstone pancreatitis found that delayed or interval procedures had significantly higher complication rates (14% and 26.5%) than the early group (4%) (p = 0.024), whereas early cholecystectomy (within 48 hours) was safe [17].

The decrease in recurring admissions in our non-surgical group following adherence to guidelines implies that systemic improvements (triage, monitoring, or, more generally, surgery) had an impact. However, the fact that some patients still experienced recurrence emphasizes the risk associated with nonoperative therapy.

To avoid delays and stop more attacks, the Academy of Medical Royal Colleges suggests index admission for laparoscopic cholecystectomy for moderate gallstone pancreatitis [3]. This is in line with the National Institute for Health and Care Excellence (NICE) guidelines [7]. It also aligns with international best practice, such as the British Society of Gastroenterology (BSG) [18] recommendations, which frequently encourage cholecystectomy for eligible patients within two weeks or during the same hospital stay. According to our statistics, recurrence was more common, and a significant percentage of patients had either no surgery or delayed treatment before the guidelines were put into effect. The timing of surgeries improved once the guidelines were put into place, and the risk of recurrence among patients who had surgery was extremely low (mean admissions ~1, no variability). This is in keeping with the expectations of the guidelines, which state that "cholecystectomy during index admission, ideally before discharge" is necessary to reduce the risk of recurrence.

Furthermore, we observe evidence in our sample that the primary benefit of guideline-based management is the prevention of repeated episodes. Our findings align with the body of research demonstrating the effectiveness and safety of early cholecystectomy.

However, the outcomes of our non-surgical group, particularly the recurrence in certain individuals, reflect practical limitations, as not all patients can have surgery due to comorbidities, patient refusal, or budget limitations. A subset of eligible patients, not all of them, are typically assumed by guidelines. These remaining non-operated circumstances may therefore be "real-world exceptions" that are acknowledged by guidelines but not entirely addressed.

Although these patients did not undergo surgery, the implementation of the guideline appears to correlate with a reduction in recurrence rates, possibly due to better triaging, improved follow-up, or selective use of non-surgical management in lower-risk patients. The drop-in recurrent admission rate (by ~11%) indicates that even among patients not operated on, overall outcomes may have improved post-guideline, possibly due to improved adherence to other supportive or interventional strategies (e.g., ERCP or close monitoring). Further investigation may be warranted to explore why these patients were not offered surgery and whether their outcomes differ significantly long-term.

Limitations

Limitations of this study include (a) the potential for missing data or misclassification of timing; (b) the single-center design and relatively small sample size, which limit generalizability; (c) uncontrolled confounders such as comorbidities, severity of pancreatitis, presence of CBD stones, or ERCP before surgery; (d) possible selection bias in the “non-surgical” group (patients unsuitable for surgery may inherently have worse outcomes); and (e) lack of long-term follow-up beyond readmissions recorded in hospital data.

## Conclusions

This study shows that better patient outcomes and fewer readmissions result from following recommendations for index admission cholecystectomy in cases with moderate gallstone pancreatitis. Moreover, recurrence rates dropped, and the number of timely surgeries rose following the implementation of the guidelines. Patients who received non-operative treatment continued to have a higher chance of recurrence. These results underline the necessity for mechanisms that facilitate prompt care and support early surgical intervention as routine practice.
